# Small-Molecule Antibiotic Drug Development: Need and Challenges

**DOI:** 10.1021/acsinfecdis.3c00189

**Published:** 2023-10-11

**Authors:** Megan Bergkessel, Barbara Forte, Ian H. Gilbert

**Affiliations:** † Division of Molecular Microbiology, School of Life Sciences, University of Dundee, Dundee DD1 5EH, U.K.; ‡ Drug Discovery Unit and Wellcome Centre for Anti-Infectives Research, Division of Biological Chemistry and Drug Discovery, University of Dundee, Dundee DD1 5EH, U.K.

**Keywords:** antimicrobial resistance, small-molecule antibiotic, antibiotic tolerance, antibiotic drug discovery

## Abstract

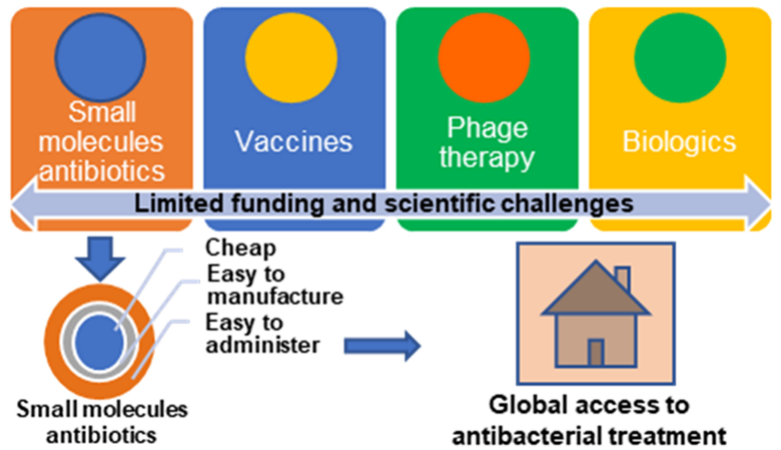

The need for new antibiotics is urgent. Antimicrobial resistance is rising, although currently, many more people die from drug-sensitive bacterial infections. The continued evolution of drug resistance is inevitable, fueled by pathogen population size and exposure to antibiotics. Additionally, opportunistic pathogens will always pose a threat to vulnerable patients whose immune systems cannot efficiently fight them even if they are sensitive to available antibiotics, according to clinical microbiology tests. These problems are intertwined and will worsen as human populations age, increase in density, and experience disruptions such as war, extreme weather events, or declines in standard of living. The development of appropriate drugs to treat all the world’s bacterial infections should be a priority, and future success will likely require combinations of multiple approaches. However, the highest burden of bacterial infection is in Low- and Middle-Income Countries, where limited medical infrastructure is a major challenge. For effectively managing infections in these contexts, small-molecule-based treatments offer significant advantages. Unfortunately, support for ongoing small-molecule antibiotic discovery has recently suffered from significant challenges related both to the scientific difficulties in treating bacterial infections and to market barriers. Nevertheless, small-molecule antibiotics remain essential and irreplaceable tools for fighting infections, and efforts to develop novel and improved versions deserve ongoing investment. Here, we first describe the global historical context of antibiotic treatment and then highlight some of the challenges surrounding small-molecule development and potential solutions. Many of these challenges are likely to be common to all modalities of antibacterial treatment and should be addressed directly.

## Introduction

Rising rates of antimicrobial resistance are widely considered a looming crisis, with the WHO declaring several bacterial pathogens to be “critical priorities”^1^ and naming antimicrobial resistance mitigations as one of five major platforms in its program of work for 2019–2023.^2^ However, consensus has not been reached on the best way forward. Since the introduction of Salvarsan for the treatment of syphilis in 1910,^3^ the dominant strategy for the treatment of bacterial infections has been the discovery and production of small-molecule antibacterial drugs. Key features of these classic small-molecule antibiotics are that they are approximately 400–1200 Da in size, chemically defined, generally inexpensive to produce, and relatively stable for storage, albeit with some exceptions. For example, more than 30 different small-molecule antibiotic drugs are included in the WHO’s list of essential medicines. Many of these drugs can be administered orally, stored at temperatures up to 25 °C, and have estimated generic production costs that are below US$ 1.00 per daily defined dose (DDD).^4^ Antibiotics on this list include: fully synthetic compounds (ciprofloxacin, trimethoprim, sulfamethoxazole, linezolid, and chloramphenicol); natural products produced by microbial fermentation (erythromycin and gentamicin); and those produced semisynthetically (azithromycin, amoxicillin, doxycycline, and clindamycin). Most newer antibiotics with improved resistance profiles are substantially more expensive, although this cost is not entirely related to the production costs. Some important classes, such as carbapenems, are notoriously unstable.^5^ Nevertheless, high efficacy, ease of use, and low cost have made small molecule antibiotic drugs a mainstay of modern medicine globally.

Some have claimed that the pipeline for new small-molecule drug development is now “broken”.^6^ As a result, the focus of antibacterial research is shifting toward entirely different approaches, including vaccine development, phage therapy, and other biologics (treatments such as monoclonal antibodies or antimicrobial peptides that are derived from biological sources and do not have defined chemical structures).^7^ Among 64 antibacterial therapies in clinical development as of 2022, 17 were biologics.^6^ For preclinical and early clinical development, the portfolio of the nonprofit consortium CARB-X (Combatting Antibiotic-Resistant Bacteria) can serve as a representative sample. Among 26 therapeutics or preventives that are actively being funded, only 7 are small-molecule drugs, compared to 31 out of 51 previously funded projects.^8^

Tackling a problem this challenging and serious requires many approaches; all possibilities should be considered, and exploration of novel approaches encouraged. However, this should not come at the expense of continued investment in the key strategy on which we have relied for the past 80 years. Small-molecule drug discovery for antibacterial therapies has been one of the most successful medical innovations in human history. The current burden of bacterial infection, by both antibiotic-sensitive and antibiotic-resistant bacteria, is heaviest on Low- and Middle-Income Countries (LMICs), where a lack of access to existing antibiotics is still a greater threat than antibiotic resistance. The large number of untreated, insufficiently treated, or unsuccessfully treated infections globally is an engine that drives dangerous evolutionary changes in pathogens. Therefore, ensuring that we have appropriate treatments to address all of the world’s bacterial infections should be a major priority. This requires cheap, effective, easy-to-manufacture, and easy-to-administer antibiotics, ideally paired with rapid and cheap point of care diagnostics to facilitate implementation of appropriate stewardship programs.

Challenges and opportunities in small-molecule antibiotic drug discovery have been extensively reviewed in many excellent recent works (for example, refs (3), (9), and (10)). Our goal here is to take a very broad overview, allowing these issues to be explored in a global context. Evaluating the past and current patterns of infectious disease burden as well as challenges and recent advances in treatment options suggests that small molecule drugs continue to be the only viable option for meeting the bulk of medical need.

## Historical Context: Small Molecule Antibiotics Have Dramatically but Unevenly Reduced the Global Burden of Infectious Disease

**Figure 1:**
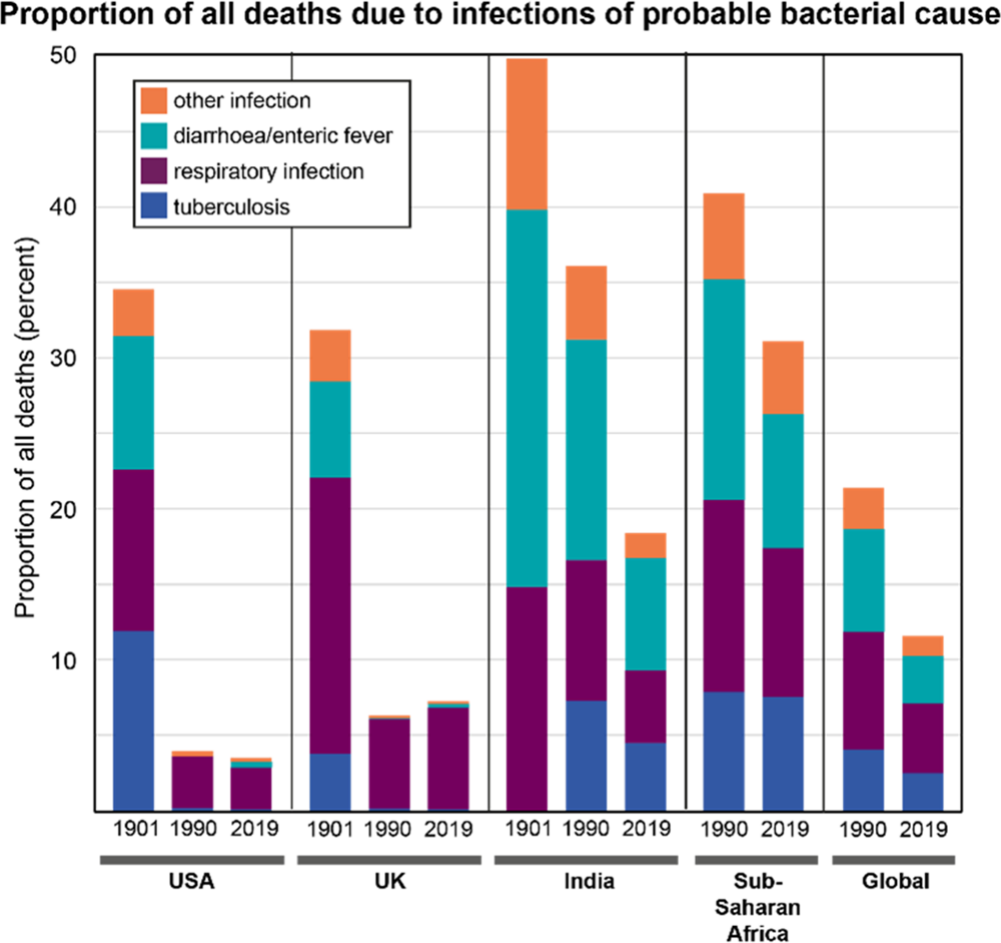
Fraction of deaths due to probable bacterial infection by year and location. Data for 1990 and 2019 were extracted from the 2019 Global Burden of Disease (GBD) study, accessed via the GBD Compare tool produced by the Institute for Health Metrics and Evaluation at the University of Washington.^11^ Infection categories were grouped as follows: “tuberculosis” included tuberculosis and HIV/AIDS-TB categories; “respiratory infection” included lower and upper respiratory infection, diptheria, and whooping cough; “diarrhea/enteric fever” included diarrheal diseases, typhoid and paratyphoid, iNTS, and other intestinal infectious diseases; “other infection” included meningitis, encephalitis, syphilis, gonorrhea, chlamydia, tetanus, maternal sepsis, neonatal sepsis, otitis media, and other unspecified infectious diseases. Data for the UK from 1901 are from the Mortality Statistics Unit of the Office of National Statistics. “tuberculosis” included ICD1 codes 460, 480, 490 and 530; “respiratory infection” included 130, 150, 360–390, and 1180; “diarrhea/enteric fever” included 180–240; and “other infection” included 80–110, 270–300, 320–350, 410, 420, 450, 630, and 830.^12^ Data for the US from 1901 were from Mortality Statistics 1900–1904.^13^ “Tuberculosis” included all tuberculosis deaths; “respiratory infection” included whooping cough, diptheria and croup, and pneumonia; “diarrhea/enteric fever” included typhoid fever and diarrhea/enteritis; and “other infection” included scarlet fever, other epidemic diseases, meningitis, and 40% of childbirth deaths.^14^ Data for India from 1901 are from “Death in India 1871–1921”.^15^ Fractions of deaths were estimates for “respiratory infection”, referring to respiratory diseases, tuberculosis, pneumonia, and bronchitis; “diarrhea/enteric fever”, referring to diarrhea, dysentery, and cholera; and “other infections”, referring to plague.

The use of antibiotics to treat bacterial infections is one of the most impactful medical interventions in human history. Antibiotics were first deployed in North America and Europe alongside improvements in sanitation and living standards that were also very important, and the effects were striking. In 1901, before the first antibiotics were available, about one-third of all deaths in the United States and United Kingdom were from (likely bacterial) infections. By 1990, these infections accounted for only about 5% of deaths (Figure 1).

In the US, deaths from infections decreased 8.2% per year from 1938 to 1952—roughly overlapping with initial clinical deployments of several antibiotics. This contrasts with decreases of only 2.8% per year from 1900 to 1938 and 2.3% per year from 1952 to 1980.^16^ At the same time, other changes to medicine, such as widespread use of chemotherapy to treat cancer, increasing prevalence of surgical interventions, and an aging population, depend on antibiotics to protect against infections that would not have been survivable a century ago.^3^

While the introduction of antibiotics helped greatly reduce the proportion of deaths due to bacterial infections, the benefits have been unevenly distributed around the world. For example, the burden of bacterial illness in India was extremely high at the start of the 20th century. By 1990, the proportion of deaths due to infection had only decreased to about the level *seen in the US and UK in 1901*. But by 2019, this proportion had further decreased by half, concomitant with a large increase in antibiotic consumption.^17^ The proportion of total deaths due to infection in sub-Saharan Africa in 2019 is *still* similar to the US and UK in 1901 (Figure 1), and antibiotic consumption rates are still relatively low.^17^

**Figure 2:**
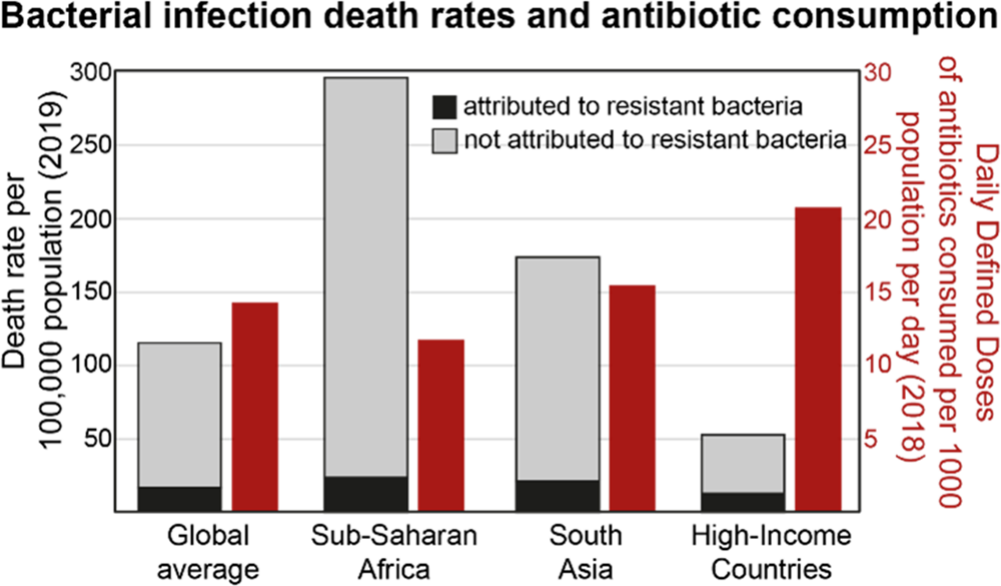
Bacterial infection death rates (2019) and antibiotic consumption (2018) for various world regions. Death rates attributed to resistant bacteria (black bars) are from ref (14). Death rates not attributed to resistant bacteria (gray bars) were calculated by adding the total death rates from 33 pathogens^19^ to the total death rates from tuberculosis (data derived from GBD2019 and retrieved from “Our World in Data” web site),^20^ and subtracting the death rate attributed to resistant bacteria.^18^ The global total death rate from 33 pathogens is not explicitly age standardized. Antibiotic consumption rates (red bars) are from ref (13).

These observations are important when interpreting data on patterns of antimicrobial resistance (AMR). A recent study estimated that globally, approximately 1.3 million deaths in 2019 were directly attributable to AMR.^18^ This number is deeply concerning and needs to be addressed urgently. However, it pales in comparison to the 8.9 million total worldwide deaths from bacterial infection that year.^11^ This difference between AMR-attributed and total deaths is especially important in LMIC regions such as sub-Saharan Africa, where death rates from AMR infections are the highest in the world, but the total burden of bacterial infection is far higher and antibiotic consumption is relatively low (Figure 2).

This point is critical: *decreasing the global burden of bacterial infections will likely require an increased use of antibiotics in parts of the world that bear the bulk of the burden*. Since the world already consumes an estimated 40 billion daily defined doses (DDDs) of antibiotics per year (as of 2018),^17^ at an estimated generic cost of approximately US$0.10 to US$1.00 per DDD,^4^ even modest increases in the cost of antibacterial therapy could have a large negative impact on the ability of patients everywhere to access treatments; our goal should instead be to improve access. Although it is important to note that more data are needed on bacterial isolate resistance rates from sub-Saharan Africa, current modeled estimates suggest that these rates are not currently substantially higher than in Europe or North America for many drug-pathogen pairs (with the exception of third generation cephalosporin-resistant *Klebsiella pneumoniae*).^18^ Increasing access to antibacterial drugs could drive increasing antimicrobial resistance, even with improved stewardship efforts, but global historical trends suggest that this is a cost worth paying for in terms of lives saved. In addition to improving access, we must prepare to meet the challenge of growing antimicrobial resistance by developing new and improved antimicrobial drugs and using them as efficiently as possible.

## How Do Antibiotic-Sensitive Bacteria Cause Death?

While much attention has been focused on developing new strategies to tackle AMR, treating infections caused by antibiotic-sensitive bacteria is an even larger unmet medical need globally. To shape strategies for addressing this need, the reasons why large numbers of deaths are currently caused by antibiotic-sensitive bacteria should be considered. Understanding these issues is important in shaping the development of new therapies.

### Lack of Access to Antibiotics

Antibiotic access is seriously lacking in many parts of the world, meaning that antibiotic treatment is never attempted for significant numbers of patients with bacterial infections. Several studies have suggested that improved access to antibiotics could save lives. For example, the MORDOR (Macrolides Oraux pour Réduire les Décès avec un Oeil sur la Résistance) trial carried out in Niger from 2014 to 2017 measured the effects on childhood mortality of mass distribution of azithromycin twice per year to all children under the age of 5. Deaths from all causes dropped by 18% in communities that received azithromycin relative to communities that received a placebo. Verbal autopsies to establish causes of death revealed that the treatment reduced deaths from dysentery, meningitis, and pneumonia (which have probable bacterial causes) as well as from malaria.^21^ The same MORDOR trial carried out in Malawi showed a 9% drop in all-cause mortality, with decreases in deaths attributed to diarrhea and pneumonia as well as to HIV/AIDS.^22^ Mass distribution of antibiotics is not a suitable long-term solution for many reasons, but these large-scale trials provide robust evidence that our existing antibiotics, despite their low cost and easy administration, are not reaching all of the people whose lives they could save.

### Antibiotic Tolerance

In all parts of the world, another cause of deaths from infections by bacteria that are classed as antibiotic-sensitive is the differential susceptibility of these bacteria to antibiotics in standardized antimicrobial susceptibility testing (AST) versus in the context of the infection. This is a particular issue in chronic infections faced by people with underlying health conditions that may compromise their ability to fight infections. For example, in people with chronic lung infections due to cystic fibrosis or non-CF bronchiectasis, results of laboratory AST correlate very poorly with clinical outcomes for a particular antibiotic.^23^ Chronic skin wounds, recurrent urinary tract infections, and infections of indwelling medical devices are other infection types that often respond poorly to antibiotic treatment, even when AST identifies drugs to which they should respond.^24,25^

The causes of bacterial tolerance of antibiotics to which they have no clear genetically determined resistance are still being actively researched. It has been proposed that stresses imposed by the immune system or exposure to other species of bacteria in a polymicrobial infection induce protective stress responses in the bacterial pathogens that can also protect against exposure to antibiotics.^26−28^ It has also been clear since antibiotics first started being used clinically that nongrowing bacteria can tolerate exposure to drugs that primarily subvert active growth processes.^29,30^ Finally, it is likely that clearance of a bacterial infection by antibiotic treatment also relies on contributions from host defenses. If these are compromised, as is the case for many vulnerable patient populations, including very young or malnourished children, then the likelihood of successful treatment is reduced. Development of new antibacterial therapeutics should actively address the existing difficulties of inadequate access in LMICs and inefficient action against chronic infections, in addition to addressing the threat of rising resistance.

## Untreated and Insufficiently Treated Infections Allow Selection for Resistance

While any death or disability caused by a bacterial infection is a tragedy, the failure to cure infections that should be susceptible to antibiotics can also contribute to the evolution of more dangerous pathogens that constitute a wider threat. This contrasts with diseases like cancer or cardiovascular disease, where a lack of access to medication is a serious problem in LMICs but the diseases cannot be directly transmitted to others. The selective pressure applied by an antibiotic is one component driving evolution of pathogen success, but other selective pressures within the host and the absolute sizes of pathogen populations also contribute.^31^ In general, persistently large pathogen populations associated with inadequately treated infections are potentially dangerous and reducing infection burden requires excellent access to effective antibiotics. An example of evolutionary pressures impacting the host range and invasiveness of a bacterial pathogen is seen in invasive nontyphoidal *Salmonella* (iNTS) isolates from Malawi. An iNTS epidemic has killed an estimated 650 000 people in sub-Saharan Africa in the past decade. Since emerging around 2007, a new lineage of the most common serotype has undergone clonal expansion and is increasing in prevalence. Interestingly, this lineage lacks some of the antibiotic resistance determinants observed in previously dominant lineages but has increased predicted invasiveness in a human host.^32^

Chronic infections that are recalcitrant to antibiotic treatment also provide dangerous opportunities for bacterial evolution, and more effective treatments for them should be a focus of future antibiotic research, as discussed further below. Patients with chronic or recurring infections often spend time receiving treatment in hospital and undergo long-term treatment with multiple antibiotics.^33^ Exposure to antibiotics under conditions promoting tolerance gives bacteria opportunities to acquire *de novo* resistance.^34^ Furthermore, it has been shown that resistance determinants can be exchanged by bacteria coexisting in hospital settings—including between different species, and even when infection control procedures are in place.^35^ It is not surprising that hospitals are often found to be hotspots for proliferation of antibiotic-resistant isolates,^36^ but this creates a dangerous situation for vulnerable patients who are hospitalised for other reasons, such as neonates or surgical patients. Reducing the burden of undertreated and chronic infections is an important part of reducing the opportunity for the evolution of increased antibiotic resistance and pathogenicity. Because of their relatively low cost to produce and administer to large numbers of patients affected by these infections, small-molecule drugs are essential tools in these efforts.

## Barriers to Small-Molecule Antibiotic Drug Development, and Possible Solutions

**Figure 3:**
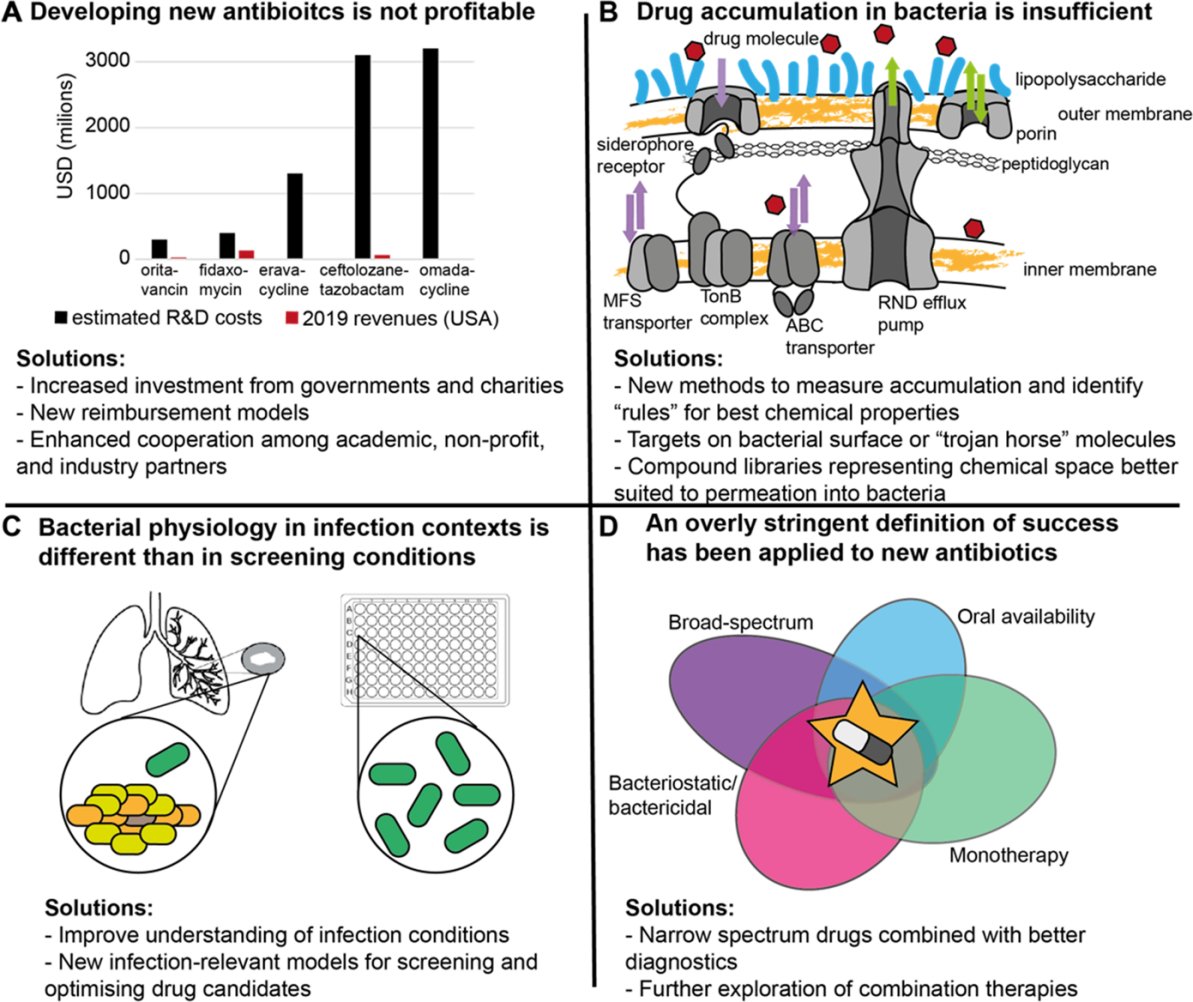
Challenges and solutions for small-molecule antibiotic drug development. (A) Costs and revenues from 5 recently approved new antibiotics. Estimated research and development costs, including an estimated share of costs for failed R&D efforts,^39^ are compared to annual revenues from US sales in 2019.^40^ For eravacycline and omadacycline revenue bars are not visible but should represent $3.3 and $8 million, respectively. Recovery of R&D costs would take many years if it were possible at all for most of these drugs. (B) Schematic of the permeability barrier in the Gram-negative cell envelope. Two lipid bilayers (yellow) are separated by a peptidoglycan cell wall, and a charged lipopolysaccharide (LPS) layer (blue) is attached to the outer membrane. Porins and RND efflux pumps are less selective (green arrows) while ABC, MFS, and TonB-dependent (siderohphore) transporters have specificity for specific substrates. To accumulate in the cell, a drug molecule must pass the charged LPS and hydrophobic lipid bilayer, then avoid efflux, or must use more specific transporters. (C) Differences in bacterial physiology between infection and screening contexts. Bacteria in infection contexts can occupy heterogeneous physiological states (mulitcolored) and often grow slowly, while traditional screening conditions produce homogeneous fast-growing populations (green). (D) Target product profiles for antibiotics have often described a “perfect” drug that is broad-spectrum, directly kills or inhibits bacterial growth, and can be administered orally as a monotherapy, which is difficult to achieve.

Many have lamented the failures of antibiotic drug development to continue production of novel effective drugs at the rate seen during the “golden age” of antibiotic research.^37,38^ Several barriers have contributed to this phenomenon, but are not insurmountable. Next, we identify some of the key issues and emerging possible solutions. These are summarized in Figure 3 and expanded in the text. Importantly, many of these barriers will also impact any other antibacterial treatment modalities, so tackling them directly will be important for all efforts to treat infection.

### The Economic Realities of the Pharmaceutical Marketplace

While scientists focused on discovering new medicines may not think of economic incentives as a fundamental problem, perverse incentives in the pharmaceutical marketplace may be the most substantial barrier faced by antibiotic drug discovery. Major pharmaceutical companies have few ongoing antibiotic development programs, and several biotech companies that have won FDA approval for new antibiotics have gone bankrupt.^41^ Simply stated, it costs far more to develop a new antibiotic than a company can hope to recover under current conditions. New drugs are reserved for stewardship purposes but also because of reluctance to use a much more expensive newer drug if an existing, inexpensive antibiotic could work. Even if new antibiotics are used, they are typically used in short treatment courses, unlike drugs for cancer, cardiovascular disease, or diabetes.^42^ The median cost (estimated in 2017) to develop a cancer drug was $640 million, and the median revenue in the first 4 years after approval was $1.7 billion.^43^ In contrast, the cost of developing a new antibiotic has been estimated at $1.5 billion, and average annual revenues postapproval at just $46 million per year.^42^ In this context, a for-profit company cannot afford to work on antibiotics.

Substantial investment by nonmarket sources–charitable foundations and governments, will be needed to support antibiotic development efforts, and new models are needed to cover the costs of drug development and manufacturing while encouraging responsible usage. Ideally, a wide range of antibiotics should be readily and globally available, and drug usage decisions should be based on fast, accurate diagnostics and clinical efficacy of the drug rather than primarily on cost. Governments have an important role to play in making this a reality, and plans for implementing subscription models of reimbursement, whereby a government pays a set price for access to a set of newly developed antibiotics regardless of the volume of use, are underway in both the UK and the US.^44,45^ Nonprofit organizations such as CARB-X in the US and GARDP in Switzerland have made important recent contributions to clinical development of new drugs.^46,47^ Improved collaboration among academic, industry, and nonprofit partners, a model that has yielded successes for drug development against neglected tropical diseases, could also provide an important path for progress.^48^ However, even after a successful drug is developed, substantial additional costs are associated with its ongoing production, distribution, and monitoring, and it is unclear whether any charitable organisations will have the funding to support this.^41^ Furthermore, the greatest need for treatment for bacterial infections is in LMICs among patients that have very limited or no means to pay for medicines. Pharmaceutical companies, while largely not pursuing antibiotic research and development themselves, have made commitments of more than $1 billion to an AMR action fund (https://www.amractionfund.com/), intended to fund work by other entities. These are promising developments, but more innovation and commitment in this area will be needed. Antibiotics must be viewed as a public good in a very interconnected world, and ensuring their discovery, production, availability, and stewardship is a priority.

### Inability of Compounds to Accumulate in Bacterial Cells

Many large-scale antibiotic discovery campaigns in the late 1990s and early 2000s were based on identifying essential genes from bacterial genomes that could be suitable drug targets and then screening large compound libraries to find inhibitors of the encoded proteins *in vitro*. Many high-potency inhibitors were identified which failed to kill bacteria because they could not access their targets,^49,50^ due to either poor penetration or rapid efflux. Additional research into the properties of bacterial cell walls and membranes has yielded insight into why they are such formidable barriers to small molecules.

Gram-negative bacteria present an especially difficult challenge as they possess two membranes, with different properties, separated by a peptidoglycan cell wall. The outer membrane is surrounded by hydrated lipopolysaccharides that repel hydrophobic molecules, while the inner membrane is a lipid bilayer. Penetration across the outer membrane may be via abundant nonspecific porins, which would favor small polar molecules, while penetration across the inner membrane could best be achieved by diffusion of a hydrophobic molecule.^51^ The nature of the cell envelope is also dynamic, with general stress responses often affecting capsule production and composition of one or both membranes.^52,53^ Finally, many bacteria possess numerous efflux systems which can effectively remove small molecules from the cytoplasm or periplasm, and which can be upregulated in response to threats.^54−56^ A successful compound against an intracellular target in a Gram-negative organism must be able to pass both membranes and evade efflux long enough to cause lethal damage, which is a small needle to thread.

Bacterial envelopes are diverse; the chemical properties needed for a compound to penetrate the cell envelope may vary substantially depending on the species of bacterium and potentially also their physiological state. Similarly, the properties of molecules required to avoid or minimize the rate of efflux are not known and will almost certainly depend on the efflux pump in question.^57^ Furthermore, the metabolism of compounds, such as β-lactams by β-lactamases, can also prevent compounds reaching sufficient levels to have a therapeutic effect.

Several strategies to overcome these difficulties are being explored. One possibility is that machine learning or AI-based approaches could be employed to predict physicochemical properties of molecules likely to have antibacterial activity.^58^ Differential killing between Gram-positive and Gram-negative species has been used as a proxy to train algorithms to predict chemical properties that affect penetration into Gram-negatives,^59^ but high-throughput methods for directly measuring compound penetration and accumulation within bacterial cells would aid these efforts. An additional consideration is that many currently used compound libraries do not lie in the most appropriate chemical space for compound accumulation in bacteria.^100^ We need further work to understand this “antibacterial” chemical space, which may vary from one pathogen to another.^57^ Novel natural products, produced by new microbial strains identified in underexploited environments, may also represent a way to identify new chemical entities with the physicochemical properties required to accumulate within bacteria and have antibacterial activity.^3^ A proviso here is that natural products rarely have suitable pharmacokinetic properties themselves, and any modification of their structure may lead to a loss of intracellular exposure; this would require careful monitoring. However, they could lend insight into suitable antibacterial targets or the chemical space for medicinal chemistry exploration.

Another approach has been to seek compounds with targets on the surface of the cell. Several recently described promising compounds have targets in the outer membranes of Gram-negative bacteria.^60^ Finally, some are exploring “trojan horse” approaches, in which receptors and transporters for required nutrients, such as iron, are subverted to facilitate antibiotic entry.^61^ A recently approved example is cefiderocol, which combines siderophore activity with a cephalosporin antibiotic, thus subverting the iron transport machinery of Gram-negative bacteria to gain access across the outer membrane.^62^ Future efforts could combine these strategies, leveraging both computational approaches and ongoing characterization of bacterial receptors, transporters, and porins to overcome the formidable cell envelope barrier.

### Neglecting Infection-Relevant Bacterial Physiology

Essentially all antibiotic drug discovery thus far has evaluated compounds based on their ability to prevent bacterial growth and/or kill bacteria that are actively growing. High-throughput phenotypic screens throughout the 1970s and 1980s repeatedly identified the same classes of compounds that effectively hit targets essential for growth.^3^ However, as discussed above, the conditions encountered by bacteria in the infection context may be different from those traditionally used to screen compounds for antibiotic activity. Growth rates, especially in chronic infection contexts, have been demonstrated to be highly heterogeneous and likely much slower than typical growth rates in the laboratory.^63,64^ Additionally, the specific stresses encountered in infections may trigger responses that are protective against antibiotics.^65^ Many efforts are underway to gain a better understanding of infection-relevant bacterial physiologies, and to design laboratory conditions for compound screening that better reproduce these physiologies.^66,67^ Phenotypic screens carried out under relevant conditions, followed by target deconvolution of hits, could reveal novel targets that are important for bacterial survival in an infection. Identification of novel infection-relevant targets could open the door for the application of modern drug development methodologies, under the umbrella of structure-based drug discovery (SBDD).^68^ These tools have already started to be applied to antimicrobial drug discovery,^69,70^ and are rapidly evolving to incorporate increasingly sophisticated analysis of multiple data types.^71^ Combination of structure-based methodologies with methods for predicting drug penetration into bacteria and measuring intracellular compound exposure, as discussed above, could dramatically accelerate compound optimization. More complex infection-relevant models, incorporating human cells, are also being explored, and could be valuable secondary screening tools.^72^ In some contexts, such as chronic infections, such models may even be able to improve upon existing animal models.^73^

### Overly Stringent Definitions of Success

Traditionally, the goal of antibiotic drug discovery campaigns has been a compound that can act alone (monotherapy) as a broad-spectrum bacteriostatic or bactericidal drug that is suitable for oral administration. These criteria have been important for drugs intended to be widely administered with easy access and minimal diagnostic burden, as antibiotics have been traditionally used. New drugs meeting these criteria would be welcome.^74^ However, especially with improvements in rapid, point-of-care diagnostic technologies, loosening some of these requirements could lead to novel treatments that are equal to or even better than existing options in some cases.

#### Combination vs Monotherapy

Although monotherapies make many steps of preclinical development and clinical trials much simpler,^75^ combination therapies have been the standard of care for many infectious diseases, such as tuberculosis, malaria, and HIV/AIDS, for decades. With a proliferation of β-lactam/β-lactamase inhibitor combinations becoming clinically available for bacterial infections, strategies for developing and evaluating combination therapies for more bacterial infections are gaining acceptance.^76^ In theory, the use of appropriate combinations of drugs could offer solutions to many pressing challenges. For example: (1) As with β-lactamase inhibitors, novel compounds could seek to alter phenotypes of the bacteria to improve efficacy of a coadministered existing antibiotic. This could include compounds that modulate efflux pump activity or outer membrane permeability in addition to β-lactamase activity.^77^ (2) Combinations could be used to address bacteria that are in different physiological states, such as actively dividing cells and tolerant bacteria in low-activity states.^78^ Such distinct subpopulations of bacteria may have largely nonoverlapping vulnerabilities and targeting them with distinct drugs is already a common strategy for treating tuberculosis.^79^ (3) Combinations may reduce the rate of resistance generation, where targets of single antibacterial drugs can rapidly acquire mutations that confer resistance. Consideration of strategies to impede the evolution of resistance will be critical for protecting existing and novel antibiotics, and drug combinations already play this role in treatment of tuberculosis, malaria, and HIV/AIDS. Clinical trials investigating the efficacy of combinations for a range of serious bacterial infections have mostly been small and inconclusive^80^ and even studies of pairwise combinations of antibiotics under growth-promoting conditions in the lab have struggled to predict whether any given combination of two antibiotics will act synergistically or antagonistically.^81^ However, hollow fiber models have identified some promising synergistic combinations for use in treating neonatal sepsis, for example.^46,82^ Ideally, synergistic combination partners should have equivalent pharmacokinetic properties and tissue distribution, and achieving this is very challenging. Laboratory or animal models with good predictive power for efficacy in humans will greatly facilitate the investigation of these issues.

#### Narrow Spectrum Vs Broad Spectrum

Another avenue for exploration is compounds that have a narrow spectrum of activity. While many currently used antibiotics are active only against Gram-positive or Gram-negative bacteria, compounds that act in a species-specific manner have not previously been pursued. However, when paired with accurate diagnostics, such compounds could allow for aggressive treatment against a pathogen while minimizing commensal microbiome disruption.^83^

#### Intravenous vs Oral Administration

Finally, although oral administration is desirable and necessary for widening access, many recalcitrant bacterial infections, especially in high-income countries, are treated in a hospital setting already, accommodating IV administration. The cost/benefit analysis of existing treatment and future research and development options will continue to change, both as new technologies emerge and as the threat of untreatable bacterial infections rises, and we must continue to re-evaluate options for taking action.

## Conclusions and Outlook

There is clearly a need for new treatments for bacterial infections. We must improve our ability to fully cure infections caused by bacteria that lack genetic resistance determinants, but can tolerate antibiotic exposure, which is currently a major contributor to the burden of bacterial illness. Additionally, the problem of ongoing selection for pathogens that are genetically resistant to existing drugs will continue to grow and at truly terrifying rates for some locations and infection types. These are interconnected problems, and solutions must consider the global context.

### Roles for Other Treatment Modalities

Some of the options currently being re-evaluated for reducing the burden of bacterial infections move away from small-molecule antibiotics completely. These include new vaccines against bacterial infection, phage therapy, and antimicrobial peptides derived from immune defenses of animals, plants, and other microbes.^7^ These options could hold promise in specific instances, but in many cases, they are likely to suffer from the same vulnerabilities as current antibiotics and depend on the continued use of antibiotics in parallel. For example, promising efforts are underway to develop vaccines against Group A *Streptococcus*,^84^*Salmonella enterica* ser. Paratyphi, pathogenic *Escherichia coli*, *Clostridium difficile*, and *Neisseria gonorrheae*, among others.^85^ Vaccines are preventives rather than treatments, and clearly both are needed to combat infectious disease most efficiently. However, vaccine and drug development efforts can compete for the same sources of funding, and it is important to consider how best to address global challenges. Vaccine development against some opportunistic pathogens, which cause infections that are difficult to treat with antibiotics in immune-compromised patients, is not considered feasible.^85^

In the case of phage therapy, a limited number of customized treatments deployed under compassionate use authorizations have been successful against infections that failed to be cleared by all available antibiotics. For extensively drug-resistant infections in high-resource settings, customized phage cocktails may increasingly become the best or only option. However, even in successful cases to date, bacterial immunity to the phages rapidly evolved, and coadministration of antibiotics was required to achieve clearance of the infection. Furthermore, these treatments are estimated to have cost on the order of 1000 times more than a course of standard antibiotics.^86^

Antimicrobial peptides are thought to be less likely to select for resistance mutations than small-molecule antibiotics, which is a desirable property in the face of rising resistance. However, they are vulnerable to degradation within a patient, increasing the difficulty of getting them to protected niches within the body and complicating PK/PD analysis. They are also costly to produce and store and subject to more complex regulatory approval than small-molecule drugs due to their status as biologics.^87^

### The Future of Small-Molecule Antibiotic Drug Discovery

There are many compelling reasons why small molecule antibacterial drug discovery should be prioritized. As most infections are found in resource-limited settings, the drugs to treat them need to be cheap to produce, stable without cold storage, and ideally orally bioavailable. Many of these requirements cannot be met by the nontraditional approaches being explored, at least with current technology. Discovery of new small molecule antibacterials has proved challenging. This can lead to the “we have tried this before” syndrome. However, learning lessons from the past provides new avenues to explore within small-molecule drug discovery. Rather than completely shifting focus to different modalities, we can overcome pitfalls^88^ with renewed commitment to innovation in this area. In most cases, barriers to development have yet to be uncovered for more complex and novel modalities. Exploration of novel modalities should continue as future success will likely require combinations of all possible solutions. However, if we take the trajectory of rising antibiotic resistance to its logical conclusion, we should assume that many of our existing antibiotics will fail in the future. We must acknowledge that the only currently feasible option for maintaining, and even improving, the degree of global access to life-saving antibacterial therapy that we have come to depend upon is to develop replacement small-molecule drugs that are similarly cheap, stable, and effective. This will be an ongoing requirement; we must continue to develop new chemical matter to stay ahead of the inevitable march of evolving resistance. Governments, research charities, and the pharmaceutical industry should invest in these efforts, concomitant with the degree to which our societies *depend* on the continued success of small-molecule antibiotic discovery.
